# Editorial: Agrochemical use impact on bee physiology

**DOI:** 10.3389/fphys.2023.1345638

**Published:** 2023-12-12

**Authors:** Yong-Jun Liu, Chunsheng Hou, Rui Guo, Eric Darrouzet

**Affiliations:** ^1^ State Key Laboratory of Resource Insects, Institute of Apicultural Research, Chinese Academy of Agricultural Sciences, Beijing, China; ^2^ Institute of Bast Fiber Crops and Center for Southern Economic Crops, Chinese Academy of Agricultural Sciences, Changsha, China; ^3^ National and Local United Engineering Laboratory of Natural Biotoxin, Fujian Agriculture and Forestry University, Fuzhou, China; ^4^ Institut de Recherche sur la Biologie de l’Insecte (UMR 7261) CNRS, University of Tours, Tours, France

**Keywords:** bee, Agrichemical, pesticide and insecticide, stressor, microbiom, sublethal activity

## Introduction

Bees, essential for pollinating crops and wildflowers, are facing a global decline. They are regularly exposed to various agrochemicals such as insecticides, herbicides, fungicides, and acaricides. As bees have limited detoxifying enzymes, they are especially vulnerable to these chemical compounds, leading to sub-lethal effects that impair their foraging and orientation abilities, with potential negative colony-level consequences. Furthermore, these agrochemicals may combine with multiple stressors such as parasites, pathogens, or nutritional deficiencies that significantly impact bee health. Therefore, it is necessary to understand the factors driving honey bee decline and to guide potential management strategies. The aim of the current Research Topic “*Adverse Effects of Agrochemicals on Bee Health*” in Frontiers in Physiology is to gain insights into the adverse impacts of agrochemicals on bee health. There are five original research articles and one review published in this Research Topic, focusing on the noteworthy influence of various pesticides and fungicides on the health, behavior, and development of bee species, including *Bombus terrestris* and *Apis mellifera*.

Neonicotinoids are the most widely used pesticides globally, which negatively impact non-target pollinator bees. Among these insecticides, thiacloprid is a commonly used compound with relatively low toxicity. The effect of sublethal larval exposure to thiacloprid on the antennal activity of adult honeybees (*A. mellifera* L.) is not yet fully understood. Ke et al. found that honeybee larvae exposed to sublethal concentrations of thiacloprid reduced the electroantennography (EAG) response in adult bees to floral odors, leading to increased olfactory selectivity in higher dosage groups. Additionally, thiacloprid negatively impacted odor-related learning and memory in emerged adult bees. Another pesticide, flupyradifurone (FPF), a systemic butenolide insecticide, has a lower binding affinity to insect nAChRs than traditional neonicotinoids and is effective against many neonicotinoid-resistant insects. Gao et al. investigated the acute toxicity of FPF on nurse bees. The authors found that nurse bees are more sensitive to FPF than larvae, with significant differences in lethal dose (LD50) values. Sublethal concentrations of FPF induced notable apoptosis in the neurons of nurse bees and altered the expression of immune-related genes.

In addition to the effects of neonicotinoid insecticides on bee health, fungicides are another major threat to bees. Xiong et al. investigated the impact of the strobilurin fungicide pyraclostrobin on the development and physiology of honeybee larvae and pupae. In the study, two-day-old larvae were exposed to field-realistic concentrations of pyraclostrobin, leading to significant decreases in survival rates, growth, delayed development of larvae, and alteration of the expression levels of several key genes related to development, nutrition, and immunity.

Bees are more likely to be exposed to multiple agrochemicals during foraging. Lu et al. examined the toxic effects of the pesticides chlorothalonil and acetamiprid on honeybee larvae. The authors identified no observed adverse effect concentrations (NOAEC) for these pesticides. The results indicated that exposure to both pesticides, even below NOAEC, significantly changed the expression of genes associated with the toxicological process in larvae, including caste development (*Tor* (GB44905), *InR-2* (GB55425), *Hr4* (GB47037), *Ac3* (GB11637) and *ILP-2* (GB10174)), immune system response (abaecin (GB18323), defensin-1 (GB19392), toll-X4 (GB50418)), and oxidative stress response (*P450*, *GSH*, *GST*, *CarE*).

Another research performed, by Tang et al. focused on the sublethal effects of spinetoram and glyphosate on physiological biomarkers and gut microbes in bumblebees (*B. terrestris*). They discovered that spinetoram and glyphosate significantly elevated superoxide dismutase activity while reducing gut α-amylase activity. However, these chemical compounds had no significant effect on glutathione-S-transferase, carboxylesterase, or gut protease activities. In addition, glyphosate increased the activity of prophenoloxidase. Moreover, they observed that glyphosate reduced the relative abundance of Zygosaccharomyces relative to fat accumulation, indicating they could affect bumblebee’s health by inhibiting energy acquisition.

Agrochemicals are often used in mixtures with spray adjuvants to enhance physicochemical properties to increase their efficacy. Nevertheless, recent laboratory studies showed that adjuvants can have a toxicity-increasing effect when mixed with insecticides. The semi-field study conducted by Wernecke et al. investigated whether organosilicon surfactants (OSS) mixed with pyrethroid and carbamate can enhance the toxic impact of insecticides on bees and colonies. Their results revealed that the insecticides, either alone or mixed with OSS, did not significantly affect bee mortality, population, and brood development, except for a decrease in flower visitation after carbamate treatments.

Gaubert et al. reviewed the current knowledge about the exposure of honeybees to various stressors and the individual and social defense mechanisms that bees have developed. Their review addresses how honeybees encounter both biotic and abiotic stressors, which can interact synergistically or antagonistically, affecting their immune and detoxification systems. This review highlights honeybees’ behavioral strategies and the production of antimicrobial compounds in host responses to these stressors.

The collective research presented in this Research Topic provides a comprehensive overview of the adverse effects of agrochemicals and other stressors on the health of larvae or adult honeybees and bumblebees under different treatments ranging from neonicotinoids and fungicides to organosilicon surfactants. Generally, the interaction of multiple stressors may be more detrimental than a single one. Consequently, it is necessary for continuous research to better understand the complex interactions of those stressors on bee physiology, behavior, and colony health. Ultimately, this knowledge will guide the development of sustainable agricultural practices that safeguard bee populations and ensure the pollination services they provide, benefiting both ecosystems and food production.

